# A guanidine-appended *scyllo*-inositol derivative AAD-66 enhances brain delivery and ameliorates Alzheimer’s phenotypes

**DOI:** 10.1038/s41598-017-14559-7

**Published:** 2017-10-26

**Authors:** Dohyun Lee, Woo-Sirl Lee, Sungsu Lim, Yun Kyung Kim, Hoe-Yune Jung, Sanket Das, Juhyun Lee, Wenjie Luo, Kyong-Tai Kim, Sung-Kee Chung

**Affiliations:** 1 Department of Life Sciences, Pohang University of Science and Technology (POSTECH), Pohang, 37673 Republic of Korea; 2 Department of Chemistry, Pohang University of Science and Technology (POSTECH), Pohang, 37673 Republic of Korea; 3 Division of Integrative Biosciences and Biotechnology, Pohang University of Science and Technology (POSTECH), Pohang, 37673 Republic of Korea; 4 Center for Neuro-Medicine, Korea Institute of Science and Technology (KIST), Seoul, 02790 Republic of Korea; 5 R&D Center, NovMetaPharma Co., Ltd., Jigok-dong, Pohang, 37666 Republic of Korea; 6 Helen and Robert Appel Alzheimer’s Disease Research Institute, Brain and Mind Research Institute, Weill Cornell Medical College, New York, NY 10065 USA

## Abstract

Alzheimer’s disease (AD) is a degenerative brain disease that destroys memory and other important mental functions but lacks efficient therapeutic agents. Blocking toxic amyloid β (Aβ) could be beneficial for AD and represents a promising therapeutic strategy for AD treatment. *scyllo*-Inositol (SI) is a potential therapeutic for AD by directly interacting with the Aβ peptide to inhibit Aβ42 fiber formation. Clinical studies of SI showed promising benefits on mild to moderate AD, however, with limitations on dosage regime. A new strategy to enhance the brain delivery of SI is needed to achieve the efficacy with minimum adverse effects. Herein, we report that a novel guanidine-appended SI derivative AAD-66 resulted in more effective reductions of brain Aβ and plaque deposits, gliosis, and behavioral memory deficits in the disease-established 5xFAD mice. Overall, our present study reveals the potential of AAD-66 as a promising therapeutic agent for AD.

## Introduction

Alzheimer’s disease (AD) is the most common type of dementia. It is prevalent worldwide with approximately 46.8 million patients, and the incidence increases rather rapidly because there is no available cure and the aging population is continuously increasing^1^. AD brains are characterized by two pathological hallmarks, amyloid β (Aβ) plaques and neurofibrillary tangles, which are tightly associated with neuronal damage and disease progression^2–4^. Preventing or blocking formation of neuronal toxic Aβ oligomers represents one of promising therapeutic strategies for AD. Among the several potential therapeutic agents for AD, *scyllo*-inositol (SI) has been reported to effectively block oligomerization of Aβ peptides *in vitro*, reduce Aβ plaques *in vivo*, and alleviate symptoms of AD in a mouse model^5–7^. It was reported that SI concentrations in the cerebrospinal fluid (CSF) and brain increased after its administration, and its preclinical data supported that a sustained elevated level of SI in the brain seemed necessary for the therapeutic efficacy^6–9^. Thus, SI has been clinically studied for the treatment of AD. The results indicated potential benefits on the earlier stage AD patients, but its clinical use was not recommended because of serious adverse effects in the higher dose groups^10,11^. The adverse effects at high dose may be attributed to the renal function overloaded with systemically increased levels of SI and suffered from hyperuricemia^11–13^. So increasing brain penetrating efficiency of SI could lower the SI dosage and improve SI efficacy for AD treatment while minimizing the adverse events.

In the present study, we have utilized our recently developed novel brain delivery system based on guanidine-rich molecular carriers to increase SI brain levels. Novel guanidine-rich molecular carriers showed excellent blood-brain-barrier (BBB) permeability and they successfully improved the efficacies of brain cancer treatment in mouse models after conjugation to several anti-cancer drugs including doxorubicin, 5-fluorouracil, and paclitaxel^14–19^. More recently, trehalose, an anti-aggregation agent on Huntington’s disease (HD), demonstrated improved BBB crossing efficiency with multiple guanidine (appendages) and increased neuroprotective effects^20^. We report herein that one of our guanidine-appended SI derivatives AAD-66, which has a good BBB crossing ability, improved efficacies on reducing brain pathologies as well as cognitive deficits in 5xFAD AD transgenic mice.

## Results

### Preparing the guanidine-appended SI derivative AAD-66

**Figure 1:**
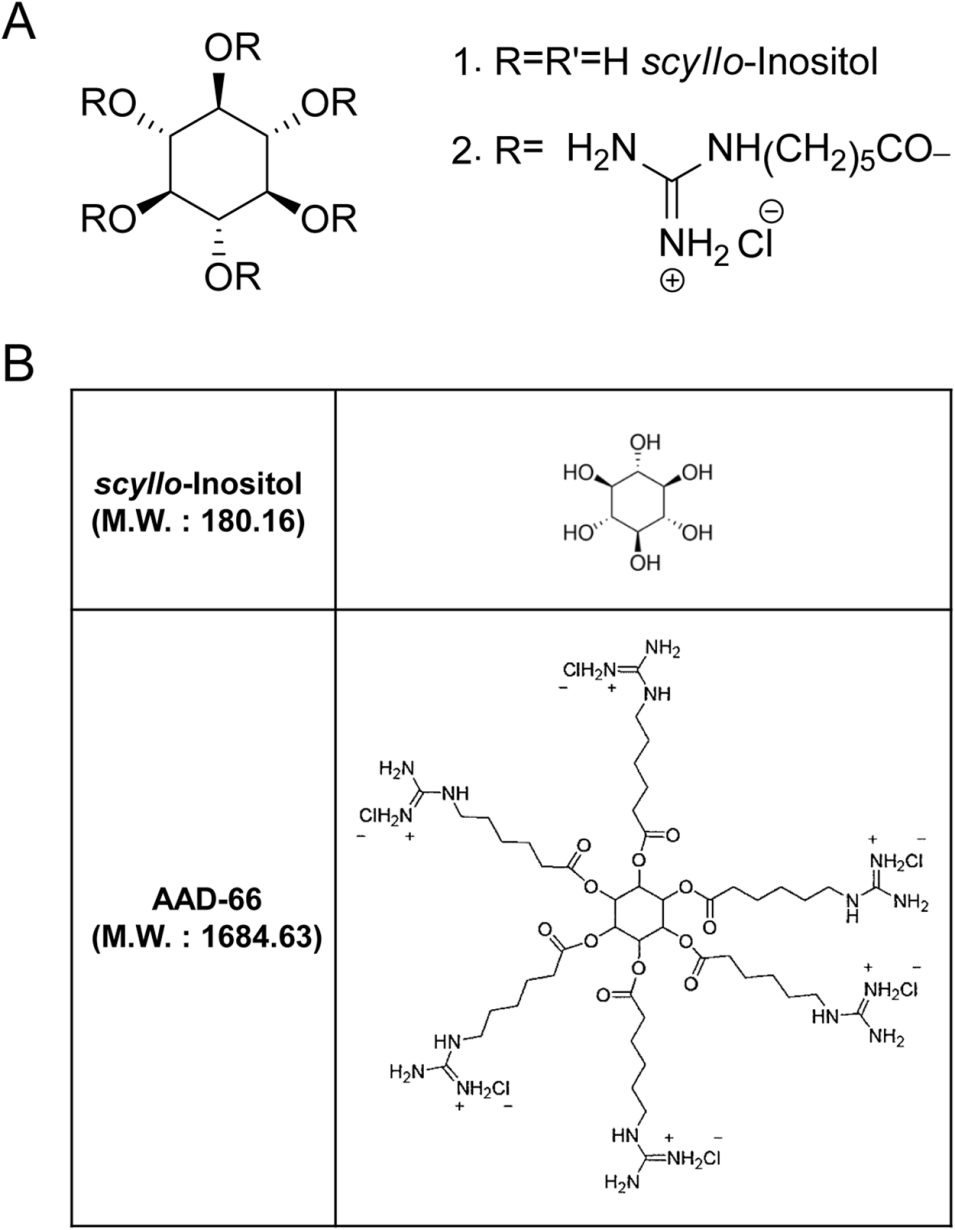
Structure of *scyllo*-inositol and its derivative AAD-66. (**A**) Schemes for the synthesis of the *scyllo*-inositol derivative AAD-66. 1, *scyllo*-inositol. 2, AAD-66. (**B**) Chemical structures of *scyllo*-inositol and AAD-66. The molecular weight of AAD-66 is about 10 times bigger than *scyllo*-inositol due to the six guanidine groups.

As guanidine-rich molecular carriers^14–17^ and the guanidine-appended modifications^20^ were found to enhance the cell membrane-crossing ability and the BBB permeability, we have prepared the guanidine-appended derivatives of SI (Fig. 1, Supplementary Methods). The synthetic design involves linking the hydroxyl groups of SI with guanidine-containing carboxylate chains via ester bonds. The ester bonds are expected to be susceptible to cleavage by intracellular esterases. The fluorescein isothiocyanate (FITC) attached SI derivatives were examined for the cellular membrane and the BBB penetration abilities. In the preliminary experiments with fluorescent microscopy and FACS analysis, AAD-56-FITC, which carries five guanidine groups together with one FITC group, showed a good efficiency in crossing cellular membranes and the BBB (Supplementary Fig. S1).

### Oral administration of AAD-66 effectively ameliorates cognitive deficits in 5xFAD mice

**Figure 2:**
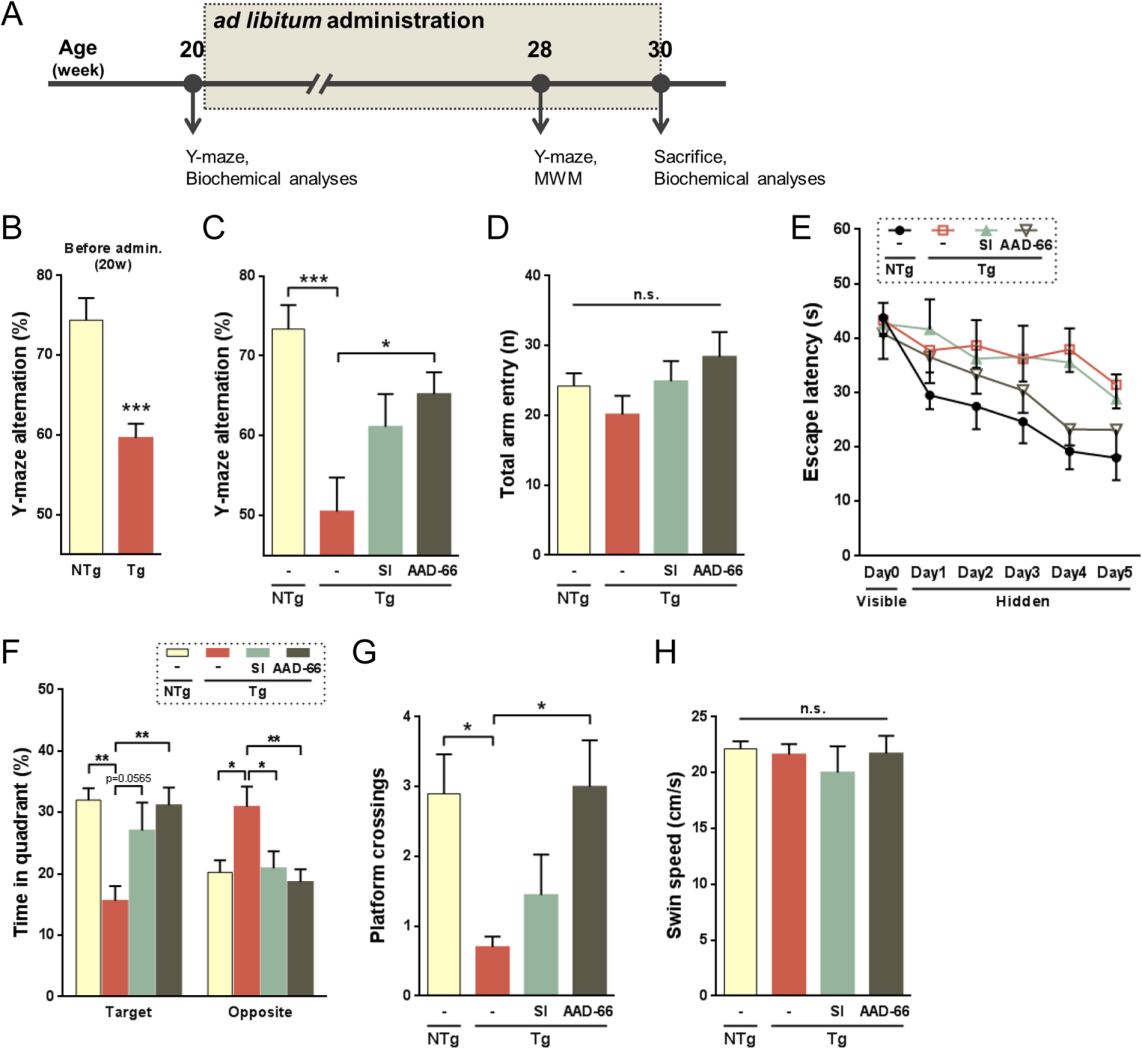
AAD-66 more effectively rescues cognitive deficits in 5xFAD mice. (**A**) Experimental time course. (**B**) Cognitive deficits before administration evaluated by Y-maze tests. Unpaired t-test, NTg (n = 10), Tg (n = 30). (**C**,**D**) Y-maze test after 10-week administration. NTg(-) n = 10, Tg(-) n = 10, Tg(SI) n = 9, Tg(AAD-66) n = 10. (**C**) Percentage of alternation on the Y-maze after administration. (**D**) Total entry number into arms on the Y-maze. (**E**–**H**) Morris water maze test after administration. NTg(-) n = 10, Tg(-) n = 10, Tg(SI) n = 9, Tg(AAD-66) n = 10. (**E**) Escape latency in the Morris water maze plotted against the training days. (**F**) Percentage of time spent in the target and the opposite quadrant during a 60 sec probe trial on the day6. (**G**) Frequency of platform crossings during probe trial. (**H**) Swim speed of the probe test. All data are presented as mean ± SEM. One-way or two-way ANOVA followed by Tukey’s multiple comparisons test. *p < 0.05, **p < 0.01, ***p < 0.001.

**Figure 3:**
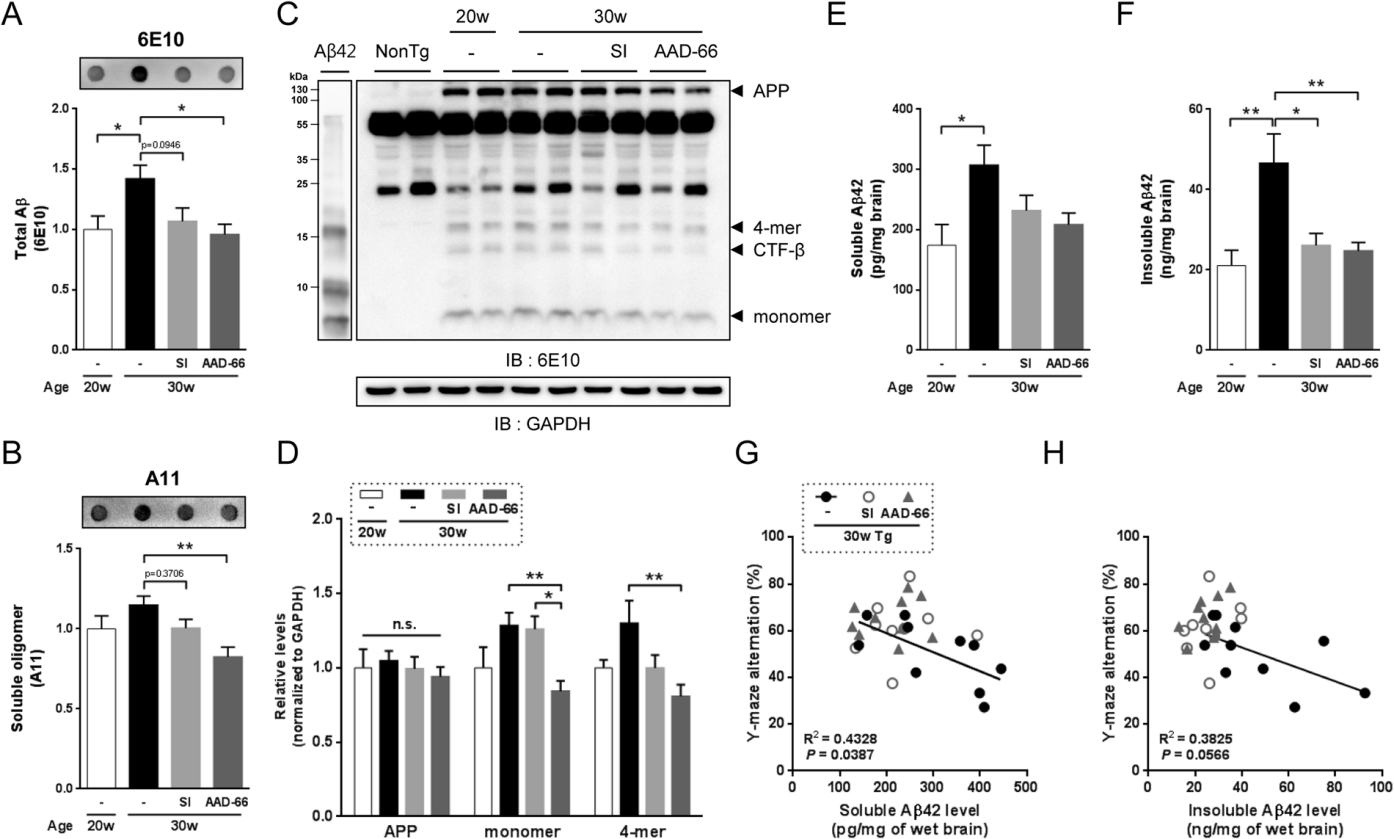
AAD-66 reduces Aβ deposit and soluble oligomers in the 5xFAD mice brain. (**A**,**B**) Total Aβ and soluble oligomers in the detergent-free TBS-soluble fractions analyzed by filter-trap assay. (**C**,**D**) Western blot analysis for APP and Aβ species. GAPDH on the same membrane was labeled after membrane stripping. Synthetic Aβ42 was used for comparing Aβ monomer and oligomer size. (**E**,**F**) Soluble and insoluble Aβ42 levels measured by ELISA. (**G**,**H**) Effect of SI and AAD-66 efficacy on the negative correlation between pathobiology and behavioral function. The solid line represents linear regression of correlations of vehicle treated group. Number of mice for biochemical analyses: pre-administration (20w) n = 8, vehicle (30w) n = 10, SI (30w) n = 9, AAD-66 (30w) n = 10. All data are presented as mean ± SEM. One-way ANOVA followed by Tukey’s multiple comparisons test. *p < 0.05, **p < 0.01.

**Figure 4:**
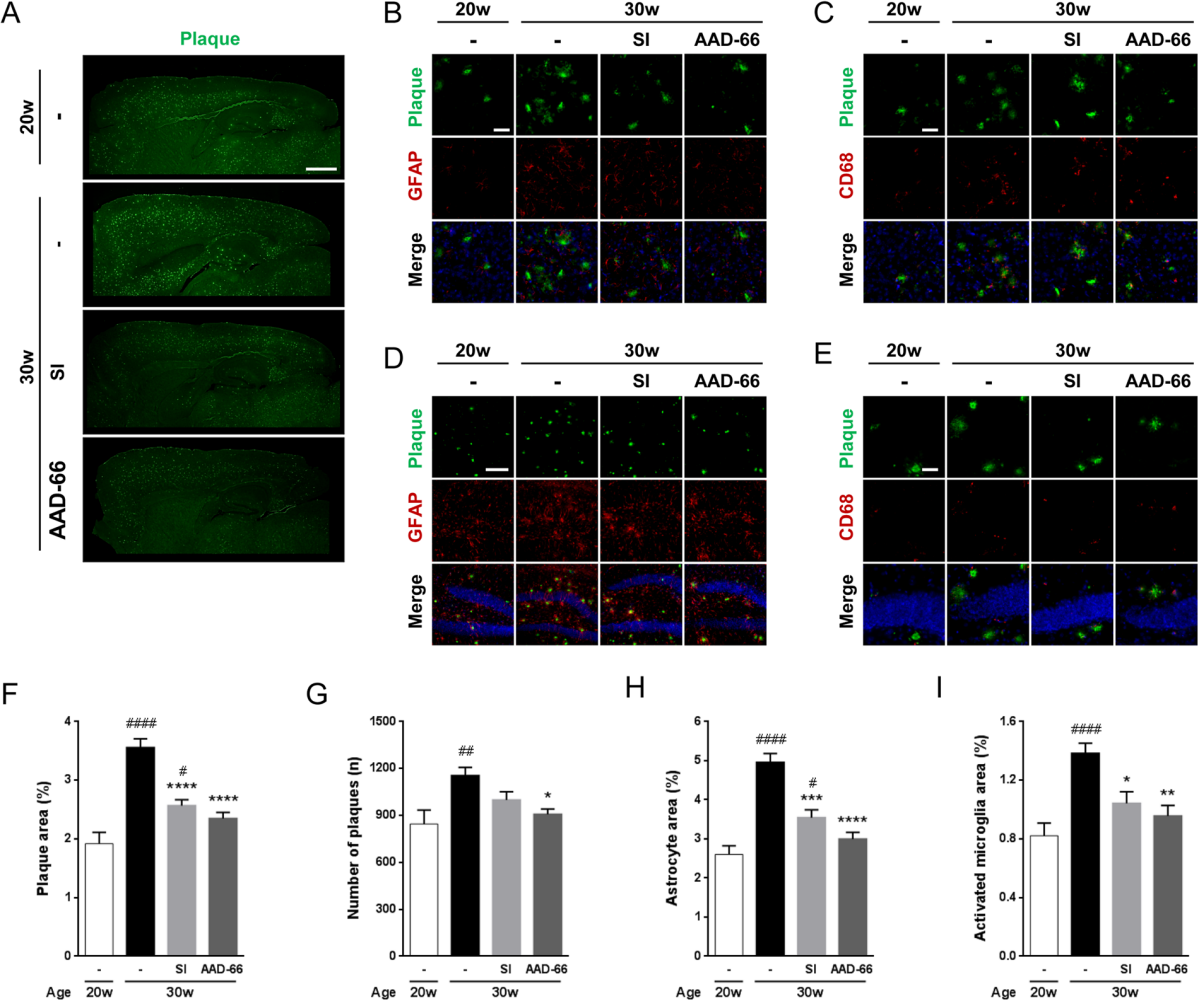
AAD-66 lowers Aβ plaque deposits and gliosis in the 5xFAD mice brain. (**A**) 6E10-labeled Aβ plaques in sagittal sections of the brains. Scale bar, 1 mm. (**B**,**C**) Astrocytes and activated microglia with Aβ plaque deposition in the cortex (images were captured at the area above hippocampus). Scale bar, 100μm. (**D**,**E**) Astrocytes and activated microglia with Aβ plaque deposition in the hippocampus (images were captured at the area of dentate gyrus). Scale bar, 400, 100μm. (**F**,**G**) Quantified Aβ plaque deposition in the entire cortex area. Plaque covered area and number of plaques. (**H**,**I**) Astrogliosis and microgliosis levels quantified by GFAP and CD68 covered area in the entire cortex. pre-administration (20w) n = 8, vehicle (30w) n = 10, SI (30w) n = 9, AAD-66 (30w) n = 10. All data are presented as mean ± SEM. One-way ANOVA followed by Tukey’s multiple comparisons test. (#) p-values compared with pre-administration 20w Tg mice. (*) p-values compared with vehicle treated 30w Tg mice. *p < 0.05, **p < 0.01, ***p < 0.001, ****p < 0.0001, ^#^p < 0.05, ^##^p < 0.01, ^####^p < 0.0001.

Oral administration of SI was previously reported to reverse the established AD phenotypes in an AD mouse model^6,7^. We, therefore, tested the efficacy of AAD-66, a SI derivative containing six guanidine groups, by oral administration at the symptomatic age of 5xFAD mice (Fig. 2A). This transgenic (Tg) mouse model expresses five human familial AD mutations, and develops amyloid deposition at 2 months of age and the behavioral memory deficits at 4–5 months of age^21^. Consistently, a spontaneous alternation deficit was detected in the 20-week-old Tg mice by Y-maze test (Fig. 2B). The Aβ deposits and gliosis were also detected in the 20-week-old Tg mice brains (Figs 3 and 4). After randomizing the 20-week-old Tg mice, AAD-66 or SI (0.3% in drinking water, 25–30mg/day/mouse) was administered orally (*ad libitum*) to the mice for 10 weeks. The spontaneous alternation task on the Y-maze was examined again at 28 weeks (Fig. 2A). We found that both AAD-66 and SI treated Tg mice showed improved performance compared with the vehicle treated Tg mice, but a better result was observed with the AAD-66 treated Tg mice (Fig. 2C). The total number of individual arm entries was not significantly changed (Fig. 2D).

To further evaluate spatial memory functions, the Morris water maze (MWM) test was performed. During the training period, the spatial learning ability was not different among the four test groups (Fig. 1E). On the 6th day, the mice were given a probe trial in order to assess the spatial reference memory. AAD-66 treated Tg mice significantly improved their working memory as measured by longer target quadrant occupancy and more target platform crossings comparable to the NonTg (NTg) control mice (Fig. 2F–H). The SI treated Tg mice also showed significant memory enhancement in the target quadrant occupancy (Fig. 2F), but the platform location crossings were not significantly rescued (Fig. 2G). Collectively, these results indicate that oral administration of AAD-66 could effectively ameliorate the established cognitive deficits in 5xFAD mice and its efficacy was better than the SI treatment.

### **AAD-66 reduces Aβ deposits in the 5xFAD mice**

After the behavioral tests described above, all mice were sacrificed at 30 weeks of age (Fig. 2A). The levels of the brain Aβ and plaque deposition were examined by biochemical extraction and analyses. SI was previously reported to reduce the amount of soluble Aβ in TgCRND8 mice brains^6^. Therefore, we firstly examined whether the soluble Aβ was also reduced by AAD-66. Detergent-free TBS-soluble fractions were analyzed by filter-trap assay using 6E10 (for the total Aβ species) and A11 (for the Aβ soluble oligomers) antibodies as previously mentioned^22^. Both AAD-66 and SI reduced the levels of total Aβ and Aβ soluble oligomers in the detergent-free TBS-soluble fraction (Fig. 3A,B). As the molecular weight of AAD-66 (1,684 g/mol) is about 10 times higher than SI (180 g/mol), the dosage of AAD-66 treated group at molar amount was about 10 times lower than the SI group, suggesting that AAD-66 has a substantial higher potency than SI (Fig. 1B).

Next, we examined the soluble oligomer species by Western blot analyses. It was found that both monomer and 4-meric oligomer species were significantly decreased by AAD-66 administration (Fig. 3C,D). The expression of the mutant human amyloid precursor protein (hAPP695) was not significantly altered by AAD-66 or SI administration (Fig. 3D). These observations indicate that AAD-66 effectively decreased the soluble Aβ species in 5xFAD mice brains to about the pre-administration (20w) levels.

Then, we further examined the soluble and insoluble Aβ42 levels by Aβ42 specific enzyme-linked immunosorbent assay (ELISA) as the 5xFAD model is known to generate Aβ42 exclusively^21^. The soluble Aβ42 levels in both AAD-66 and SI treated groups compared with the vehicle treated group showed a decreasing trend (p = 0.0692, p = 0.2451, respectively) (Fig. 3E). However, insoluble Aβ42 levels were significantly reduced by both AAD-66 and SI administration (Fig. 3F). These results are consistent with the previous report showing that SI resulted in a significant reduction in the insoluble Aβ42 levels, while the soluble Aβ42 levels did not show much reduction in the brains of the disease-established TgCRND8 mice^6,7^. Overall, our data suggest that both AAD-66 and SI caused a reduction of the Aβ42 levels and AAD-66 demonstrated a higher Aβ42 reducing potency compared to SI.

In addition, we examined the relationship between pathobiology and behavior function. The Aβ42 levels in the vehicle treated group negatively correlated with the alternation scores in Y-maze analysis (Fig. 3G,H). However, the SI or AAD-66 treated group did not show the correlation. It suggests that the treatment with SI or AAD-66 affected AD pathology in treated mice.

### AAD-66 attenuates Aβ plaque accumulation and gliosis in the 5xFAD mice

We subsequently examined Aβ plaques in these treated Tg mice brains by immunohistochemistry using 6E10 antibody. A substantial increase of plaque deposition was observed from 20 weeks to 30 weeks of age as measured by the brain area covered by Aβ plaques and the total plaque numbers (Fig. 4A–G). AAD-66 treatment resulted in a significant reduction in both the brain area covered by Aβ plaques and the total plaque numbers (34% and 21%, respectively) compared with the vehicle treated Tg mice, reaching to a similar pathological condition as 20 weeks of age (Fig. 4F,G). These results demonstrate that AAD-66 administration successfully attenuated the increase of plaque number and accumulation in the diseased Tg mice brains.

It is generally known that the Aβ deposition accompanies aberrant inflammatory functions of astrocytes and microglia^23^. Aβ oligomers or aggregates in AD brains primarily bind to several receptors such as toll-like receptors (TLRs) of astrocytes and microglia, and subsequently activate them^24–27^. These reactive responses result in abnormal proliferation or hypertrophy of glial cells, which is called gliosis^28–30^. SI was reported to reduce gliosis levels significantly in TgCRND8 mice^6^. Therefore, we examined astrogliosis and microgliosis by immunohistochemistry using GFAP and CD68 antibodies, respectively. At 30 weeks of age, both astrogliosis and microgliosis were dramatically increased in mice brains compared with the 20-week-old age group, but the increments were significantly diminished by AAD-66 and SI administration (Fig. 4B–E,H,I), likely resulting from an attenuated plaque accumulation in the diseased brains. The effect of AAD-66 was again found to be substantially stronger than with SI.

## Discussion

The preclinical and clinical studies of SI showed its potential benefits as a therapeutic agent for mild to moderate AD^6,7,10,11^. However, it was not approved for clinical use because of serious adverse effects and limited window of the dosage regime^10,11^. In the phase II clinical study, the administration of higher doses increased the CSF/brain concentrations of SI to the levels that were effective in animal models of AD, but the patients could not tolerate the doses^10,11^. The exact nature of the adverse effects has not been clearly defined, but the increased serum SI level is suspected to cause a dose-dependent decrease in uric acid, which indicates a renal function decline^10,11^. Thus, the clearance of systemically elevated SI might be impaired, and hyperuricemia might have occurred^11,31^. Hyperuricemia has been shown to represent a risk factor for exacerbating certain diseases such as gout, renal disease, cardiovascular disease, metabolic syndrome, urate lithiasis, atheroscleropathy, and hypertension. It was therefore suggested that the patients treated with higher concentrations of SI could be vulnerable to these disorders^11^. We have hypothesized that if the delivery efficiency of SI into the brain could be enhanced by applying our guanidine-appended modification strategy, we might be able to expect a good therapeutic efficacy even at lower concentration levels, thus minimizing the adverse effects.

The present study shows that the oral administration of AAD-66 significantly reduced AD-like cognitive deficits and neuropathology in the disease-established 5xFAD mice. A preliminary study carried out with a different AD mouse model, Tg2576 showed comparable results to a key portion of the present results. When 9 months old Tg mice were treated with AAD-66 (25–30 mg/day/mouse) *ad libitum* for 3 months, the hippocampal Aβ40 and Aβ42 were decreased by 37, 26, 44, and 32% for Aβ40 (soluble and insoluble) and Aβ42 (soluble and insoluble), respectively, compared with the vehicle treated Tg2576 mice (Supplementary Table S1).

AD therapies are expected to require a long-term treatment with therapeutic agents. In this connection, the toxicity profile of the therapeutic agent may be a critical consideration in evaluating therapeutic potentials. We have carried out in a preliminary fashion some toxicology studies of AAD-66 in terms of cytotoxicity in a number of cell lines, cytochrome P450 (CYP) activity, cardiac hERG K^+^ channel binding, and single dose *in vivo* toxicity, and also plasma protein binding rate and metabolic stability, which showed no unusual features (Supplementary Tables S2–S5 and Supplementary Figs S2 and S3). The AAD-66 concentration levels used in the present study, 30mg/day/mouse and 10 μM for cells, were well below the levels at which the toxicity studies were undertaken. In the Tg mice study, each mouse was given the same weight amount of SI or AAD-66. The molecular weight of AAD-66 is about 10 times higher than that of SI due to the guanidine-appended modifications (Fig. 1B). Thus, the in-take molar quantity of AAD-66 was about 10 times lower than that of SI, although substantially improved efficacies were generally observed for AAD-66 on the AD phenotypes compared with SI itself.

The results obtained in the present study are in general accord with the previous studies on the SI effects in TgCRND8 mice^6,7^. The mode of action of AAD-66 may be presumed to be similar to that of SI, although it is not clear at the moment whether the observed beneficial activity is due to the intact AAD-66 molecule or its various hydrolyzed products, structurally resembling SI. Overall, the present study shows that substance AAD-66 can inhibit plaque growth or formation possibly by interfering with growth of aggregates and fibers, or by cap-off the growing edges of the Aβ aggregates as SI has been proposed to do^7^.

A sustained elevated level of SI in the brain was proposed to be beneficial and necessary for the AD treatment^6,7^. A significant amount of SI is not likely to cross the BBB or cellular membranes by a diffusion process based on its chemical structure. The observation that the SI level in the brain is 100 times higher than in the periphery, indicates an active transport for SI in the brain must be involved^32^. Recently, two transporters, SMIT-1 and SMIT-2, have been identified to transport *scyllo*-inositol into the brain^33,34^. As the levels of transporters are not altered with AD pathology^33^, achieving the effective levels of SI in the brain was possible only with high doses in the phase II clinical study. Thus the dilemma is that the high doses are necessary but it causes serious adverse effects^10,11^. It implies that the delivery efficiency into the brain could be a critical issue when patients are treated with SI. Thus, we suggest that one way of overcoming the delivery problem of SI is to utilize a viable delivery system, in particular, the BBB-permeable guanidine-appended modifications as demonstrated in the present study. Although there might be other possible strategies to overcome the BBB, at the present time our simple strategy appears practical and should find some utilities in combating AD. In conclusion, further development of AAD-66 as a therapeutic agent for AD would be potential and promising.

## Methods

### Synthesis of *scyllo*-inositol derivatives

Chemical synthesis methods are included in Supplementary Methods.

### Mice

Generation of 5xFAD mice (Tg6799) was previously described^21^. Briefly, 5xFAD mice have transgenes that include five familial Alzheimer’s disease (FAD) mutations including the K670N/M671L (Swedish), I716V (Florida), and V717I (London) mutations in the 695 amino acids isoform of the human amyloid precursor protein (APP695), as well as M146L and L286V mutations in human presenilin-1 (PS1). The transgenes are under the control of the murine Thy-1 promoter. The male transgenic mice were bred with B6/SJL hybrid female mice and the genotypes were determined by PCR analysis of tail cut samples. The nontransgenic littermates were used as control animals and only male mice were used in this study.

Approval of the study protocol was obtained from the Pohang University of Science and Technology Institutional Animal Care and Use Committee (POSTECH IACUC) (Approval ID: POSTECH-2016–0026). All animal experiments were carried out according to the provisions of the Animal Welfare Act, PHS Animal Welfare Policy, and the principles of the NIH Guide for the Care and Use of Laboratory Animals. All mice were maintained under conventional conditions at POSTECH animal facility under institutional guidelines.

### Oral Administration

*scyllo*-Inositol (TCI, I0631) or AAD-66 in drinking water was freely administered *ad libitum* to 20-week-old 5xFAD Tg mice for 10 weeks. Saccharin salt in water (1%) was used for vehicle to mitigate the bitterness of AAD-66. The 1% saccharin-treated NTg (n = 10) and Tg (n = 10) mice were used for the control groups. *scyllo*-Inositol (0.3%) or AAD-66 (0.3%) in 1% saccharin solution was used for experimental groups (n = 9, n = 10, respectively). Water consumptions were monitored every couple of days and the drug containing waters were exchanged freshly every four to five days. Drug concentrations in the drinking water were recalculated and set according to the monitored water consumption.

### Y-maze test

The Y-maze apparatus has V-shaped three arms (40 × 3 × 12 cm) at 120° angles from each other. For the spontaneous Y-maze alternation test, mice were placed at the end of one arm and allowed to freely explore the maze during 8 min under the dim light conditions (50 lux). If the mouse has an intact short-term spatial working memory, it would show a tendency to enter the arm that is different from those recently visited. Alternation was defined as a consecutive entry in three different arms. The number and the sequence of individual arm visits were recorded and alternation percentage was calculated.

### Morris water maze (MWM)

The water maze tank (120 cm diameter) was filled with water. White non-toxic tempera paint was used to make the water opaque. Four different visual cues were seen each cardinal points. The water temperature was set around 22 °C before the experiments. Four trials (60 sec each, 30 min intervals) were performed per day during the training. The visible platform test was performed on Day 0 and the hidden platform training was performed for five consecutive days. The probe trial was performed on Day 6. The trajectories were recorded with a video tracking system (SMART v2.5, Panlab).

### Protein extraction

To better identify proteins of interest, we used three different extraction protocols serially to isolate proteins. First, the hemi-brain samples were weighed and homogenized at 100 mg/ml in detergent-free TBS with protease inhibitor cocktail (Sigma, P8340). The homogenates were divided into two tubes, one for the detergent-free TBS-soluble fraction for filter-trap analysis and the second one for the serial extraction.

To prepare the detergent-free TBS-soluble fraction for the filter-trap analysis, the detergent-free TBS homogenates were centrifuged at 15,000 rpm for 30 min at 4 °C. The supernatants were used immediately.

To prepare the soluble fraction for the Western blot analysis and ELISA, the detergent-free TBS homogenates were sonicated and centrifuged at 15,000 rpm for 1hr at 4 °C. The supernatants were stored at −80  °C until further use. The pellets were reserved for the extraction of insoluble proteins.

To prepare the insoluble fraction for ELISA, the pellets obtained above were dissolved in 70% formic acid and vigorously shaken until all pellets got dissolved completely. After centrifugation at 15,000 rpm for 1hr at 4 °C, the supernatants were stored at −80 °C until further use. Before analysis, the insoluble fraction samples were neutralized by adding 1:20 volume of neutralization buffer (1 M Tris, 0.5 M NaH_2_PO_4_).

### Filter-trap analysis

The detergent-free TBS-soluble fraction samples for the filter-trap analysis (4 μg) were loaded on nitrocellulose membranes using the Bio-Dot^®^ microfiltration apparatus (BIO-RAD) and filtered under vacuum. The membranes were dried and followed by blocking with 10% skim milk. Primary antibodies, A11 (1:2,000, AHB0052), 6E10 (1:2,000, SIG39320), and secondary antibodies (1:5,000) were used for detecting soluble oligomers and total Aβ, respectively.

### Western blot analysis

The soluble fraction samples (30 μg) were separated by SDS-PAGE on 16% Tricine gels and transferred to nitrocellulose membranes (200 mA constant for 2hr, wet transfer). The membranes were labeled with primary antibodies, 6E10 (1:1,000, SIG39320), GAPDH (1:2,000, MAB374), for overnight at 4 °C and then labeled with the HRP-conjugated secondary antibodies (1:5,000). Chemiluminescence images were captured by ImageQuant LAS 4000 (Fuji).

### ELISA

Commercially available Aβ42 sandwich ELISA kits (Invitrogen, KHB3442) were used for analyzing the soluble and insoluble Aβ42 levels. The soluble fraction samples were diluted in the ratio of 1:200 and the insoluble fraction samples were firstly neutralized using neutralization buffer in the ratio of 1:20 and diluted further to in the ratio of 1:500 in the ELISA diluent buffer. The experiment was performed as the manufacturer’s protocol. Synthetic Aβ42 peptide in the assay kit was used for standard curve (y = 67.626x^2^ + 240.72x + 4.8938).

### Immunohistochemistry

Sagittal cryo-sections (20 μm) of the perfused mice hemi-brains were used for immunohistochemistry. Antigen retrieval was performed by boiling for 20 min in sodium citrate buffer (10 mM sodium citrate in H2O, pH 6.0). For Aβ plaque, the sections were additionally soaked in 88% formic acid for 5 min. The sections were blocked with a blocking solution (5% FBS, 3% BSA, 0.3% Triton X-100 in PBS) for 1hr at room temperature, and then labeled with primary antibodies, 6E10 (1:300, SIG39320), GFAP (1:500, ab7260), CD68 (1:200, ab125212), in the blocking solution overnight at 4 °C. The sections were labeled with Alexa-488 or -594 linked secondary antibodies (1:1,000) for 1hr at room temperature followed by nucleus stain with Hoechst (1 μg/ml) for 10 min. The sections were finally mounted with Dako fluorescence mounting medium.

Imaging was performed using Nikon and ZEISS fluorescent microscopes. The area of Aβ plaques and gliosis in the entire cerebral cortex were analyzed by the “Analyze Particles” function of ImageJ program. Evenly spaced 8–10 sections per mouse were analyzed for Aβ plaques and 5–6 sections per mouse were analyzed for each gliosis. The average percentage of the analyzed area was calculated.

### Statistical Analysis

All statistical analyses were performed using GraphPad Prism version 6.0. The significance of differences was assessed by the unpaired Student’s t test or one- or two-way ANOVA followed by the Tukey’s multiple comparison tests. A p-value of p < 0.05 was considered to represent a significance. All data are presented as mean ± SD or SEM.

## Electronic supplementary material

Supplementary Information
