# Supplementary material for: A guanidine-appended scyllo-inositol derivative AAD-66 enhances brain delivery and ameliorates Alzheimer’s phenotypes
Source: Sci Rep. 2017 Oct 26;7:14125. doi: 10.1038/s41598-017-14559-7 (PMC5658413; doi:10.1038/s41598-017-14559-7)
Supplement: Supplementary file 1 — Supplementary Information [file 41598_2017_14559_MOESM1_ESM.pdf]

A guanidine-appended *scyllo*-inositol derivative AAD-66 enhances brain delivery and ameliorates Alzheimer's phenotypes

Dohyun Lee[1], Woo-Sirl Lee[2], Sungsu Lim[4], Yun Kyung Kim[4], Hoe-Yune Jung[3,5], Sanket Das[2], Juhyun Lee[3], Wenjie Luo[6], Kyong-Tai Kim[1,3,*] , Sung-Kee Chung[2,*]

[1]Department of Life Sciences, Pohang University of Science and Technology, Pohang, 37673, Republic of Korea

[2]Department of Chemistry, Pohang University of Science and Technology, Pohang, 37673, Republic of Korea

[3]Division of Integrative Biosciences and Biotechnology, Pohang University of Science and Technology, Pohang, 37673, Republic of Korea

[4]Center for Neuro-Medicine, Korea Institute of Science and Technology (KIST), Seoul, 02790, Republic of Korea

[5]R&D Center, NovMetaPharma Co., Ltd., Jigok-dong, Pohang, 37666, Republic of Korea

[6]Helen and Robert Appel Alzheimer's Disease Research Institute, Brain and Mind Research Institute, Weill Cornell Medical College, New York, NY 10065, USA

*Corresponding authors. ktk@postech.ac.kr or skchung@postech.ac.kr

Supplementary Information

Supplementary Figures and Tables

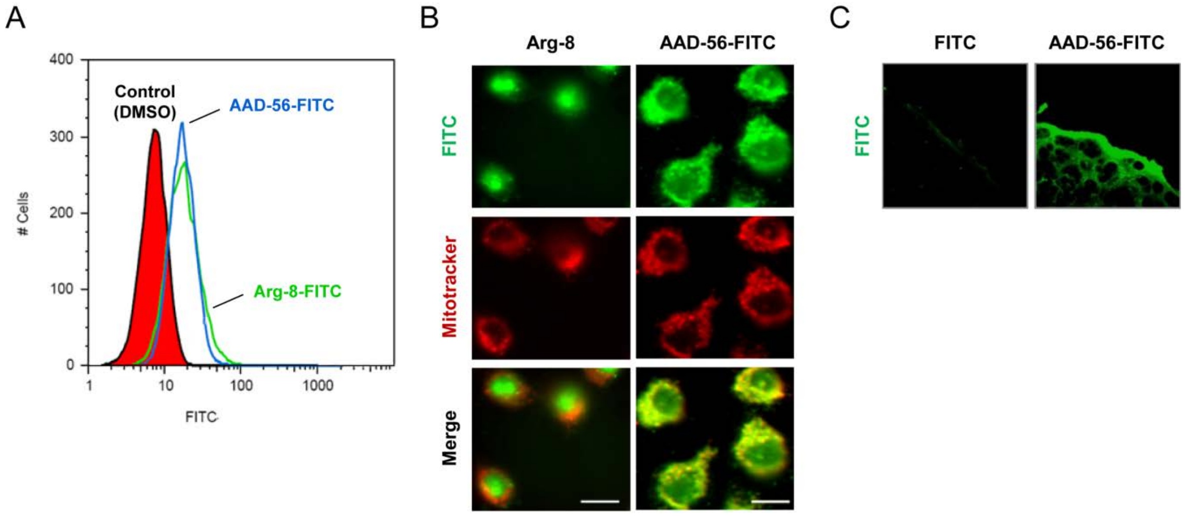

Supplementary Fig. S1. Cell membrane and BBB permeability of AAD-56-FITC.

(A) Cellular uptake of AAD-56-FITC and Arg8(positive control) in HeLa cells. Cells were treated with 5μM of each compound for 10min and analyzed by FACS. Total 10,000 cells were counted. (B) Fluorescence images of cellular uptake of AAD-56-FITC and Arg8 in HeLa cells. 10μM of each compound and 50nM of Mitotracker were treated for 1hr at 37℃. Scale bar, 20 ㎛. (C) Fluorescence images of mouse brain sections. Brain was isolated 20min after *i.p.* injection. Exposure times: 10,000ms, λmax=488nm (green fluorescence from FITC).

Supplementary Table S1. Aβ reducing effects of AAD-66 in Tg2576 mice.

	hippocampal Aβ40 (pmol/g wet brain)				hippocampal Aβ42 (pmol/g wet brain)			
	Soluble	SEM	Insoluble	SEM	Soluble	SEM	Insoluble	SEM
Vehicle	23.0	5.11	125.6	20.10	5.94	1.27	73.17	12.46
AAD-66	14.6	2.18	92.7	29.67	3.32	1.33	49.07	13.89
P-value	0.1736		0.3833		0.1836		0.2257	

9 months old Tg2576 mice (each n=6) were treated *ad libitum* with either 1% saccharin (vehicle) or AAD-66 (25-30 ㎎/day/mouse) in 1% saccharin for 3 months. Hippocampus was extracted with 2% SDS for soluble Aβ and 70% formic acid for insoluble Aβ sequentially. Aβ40 and Aβ42 levels were analyzed with ELISA assays.

Supplementary Table S2. Cytotoxicity of AAD-66 in various cell lines.

Cell line	NIH-3T3	MCF-7	PC-3	HT-29	HepG2
EC_{50} of AAD-66 (μM)	50	7.2	7.9	15.7	29.5

Cytotoxicity was examined with CCK-8 assay, NIH-3T3: mouse embryonic fibroblast, MCF-7: Breast cancer cell, PC-3: Prostate cancer cell, HT-29: Colon cancer cell, HepG2: Liver cancer cell.

Supplementary Table S3. Cytochrome P450 (CYP) activity of AAD-66 at 10μM.

	CYP1A2	CYP3A4	CYP2C9	CYP2C19	CYP2D6
AAD-66 (% of control)	110.55	102.58	92.61	83.60	43.21
Inhibitor (% of control)	2.25 (a-naphthoflavone)	2.90 (Ketoconazole)	-21.12 (Sulfaphenazole)	34.72 (Ketoconazole)	21.38 (Quinidine)

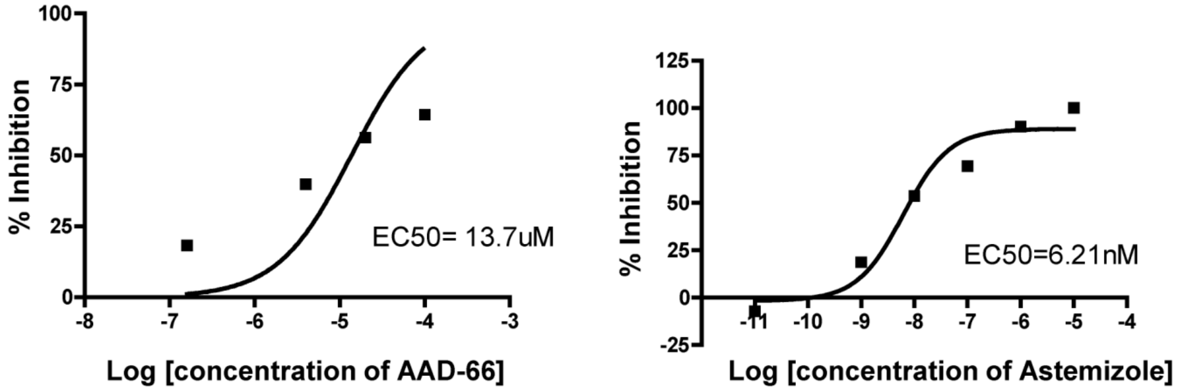

Supplementary Fig. S2. Cardiac hERG K^+ channel binding assay.

EC50 of AAD-66 was 13.7μM and EC50 of Astemizole(positive control) was 6.21nM.

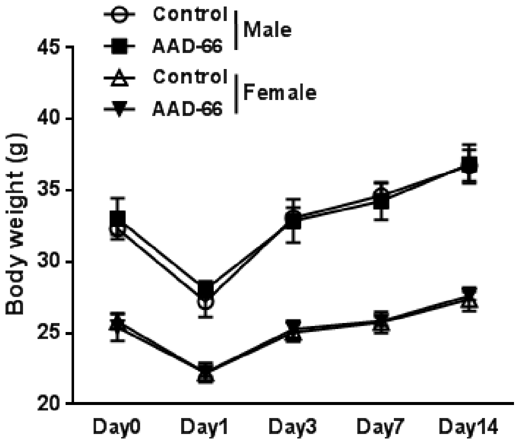

Supplementary Fig. S3. Body weight change during single dose toxicity test.

8 weeks old ICR mice (each n=5) were administered 1,000mpk of AAD-66 on day0. Body weight was measured on dya1, 3, 7, and 14.

Supplementary Table S4. Plasma protein binding rate and metabolic stability during 30min in rat plasma.

	Protein binding (%)	Plasma stability (%)
AAD-66	17.6 ± 1.5	>99

Supplementary Table S5. Human and rat liver microsomal stability (% remaining during 30min).

	Human (%)	Rat (%)
AAD-66	>99	>99

Supplementary Table S6. List of antibodies

Primary antibodies	Supplier	Catalog No.	Species / Type	Dilution
Oligomer A11	Thermo	AHB0052	Rabbit polyclonal	1:2,000
β-Amyloid, 1-16 (6E10)	Covance	SIG-39320	Mouse monoclonal	1:2,000 for Dot-blot<br>1:1,000 for WB<br>1:300 for IHC
GAPDH	EMD Millipore	MAB374	Mouse monoclonal	1:2,000
GFAP	Abcam	ab7260	Rabbit polyclonal	1:500
CD68	Abcam	Ab125212	Rabbit polyclonal	1:200
**Secondary antibodies**	**Supplier**	**Catalog No.**	**Target species**	**Dilution**
Anti-Rabbit IgG (H+L), HRP Conjugate	Promega	W401B	Rabbit	1:5,000
Anti-Rabbit IgG (H+L), HRP Conjugate	Thermo	31430	Mouse	1:5,000
Alexa Fluor 594 goat anti-rabbit IgG (H+L)	Invitrogen	A11012	Rabbit	1:1,000
Alexa Fluor 488 goat anti-mouse IgG (H+L)	Invitrogen	A11001	Mouse	1:1,000

Supplementary Methods

1. Synthetic Part

Scheme 1 Synthesis of FITC labeled *scyllo*-Inositol-G5 (AAD-56-FITC) (**2**)

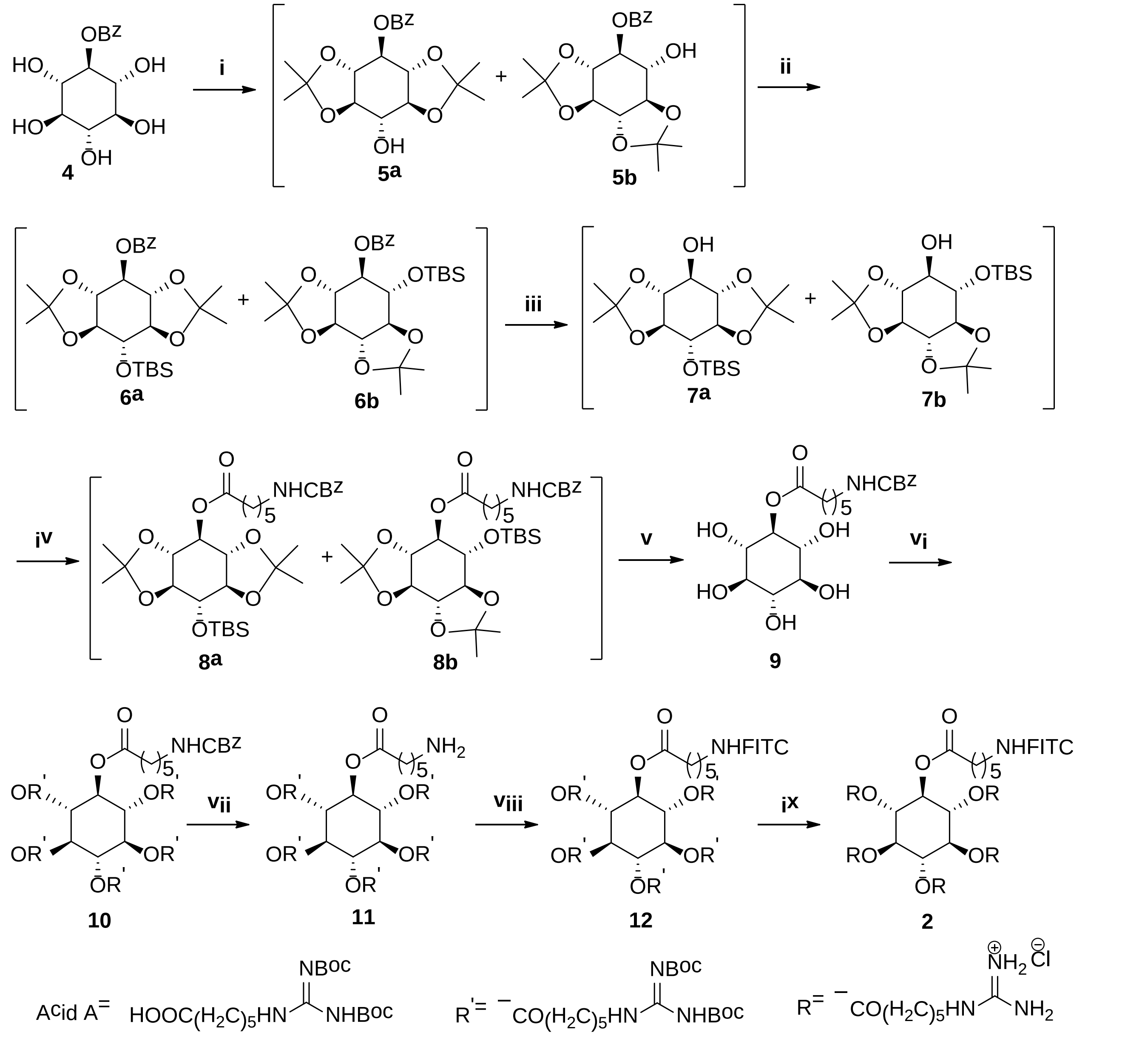

i) 2-methoxypropene, p-TSA, DMF, 81%; ii) TBDMSCl, imidazole, DMF, 88%; iii) NaOMe, MeOH-DCM, reflux, quantitative; iv) 6-benzoyloxycarbonyl-aminohexanoic acid, EDC, DMAP, DCM, 81%; v) HCl(g), EtOAc, MeOH-DCM, 91%; vi) Acid A, EDC, DMAP, DMF, 91%; vii) 10% Pd/C, H_2 (1atm), MeOH-DCM, 92%; viii) FITC-I, Et_3N, EtOH-THF, dark; ix) HCl(g), EtOAc, rt, 68% over two steps;

General Methods.

All non-hydrolytic reactions were carried out in oven-dried glassware under an inert atmosphere of dry argon or nitrogen. All commercial chemicals were used as received except for solvents, which were purified and dried by standard methods prior to use. Analytical TLC was performed on a Merck 60 F254 silica gel plate (0.25mm thickness) or analytical reverse-phase TLC on a Merck RP-8 F254s, and visualization was done with UV light (254nm and 365nm), and/or by spraying with a 5% solution of phosphomolybdic acid or ninhydrin, followed by charring with a heat gun. Column chromatography was performed on Merck 60 silica gel (70-230 or 230-400 mesh), and MPLC was performed on Fluka 100 C8-reversed phase silica gel. Melting points were determined on a Thomas-Hoover MP apparatus and uncorrected. NMR spectra were recorded on a Bruker DPX 300 (^{1}H-NMR at 300MHz; ^{13}C-NMR at 75 MHz) or Bruker DRX 500 (^{1}H-NMR at 500MHz; ^{13}C-NMR at 125MHz) spectrometers. Tetramethysilane was used as reference, and the chemical shift were reported in ppm and the coupling constant in Hz. Analytical HPLC was performed on Agilent 1100-HPLC Chemstation with an analytical column ZORBAX SB-C8 (5μm, 4.6mm ID x 25cm) or KROMASIL 100-5C8 (250 x 4.6 mm, ID: 10 mm). Low resolution mass spectra were determined on a Micromass PLATFORM ll (EI and FAB). High resolution mass spectra were obtained on JMS-700 or MALDI TOF mass spectra on a Voyager-DE STR system at the Korea Basic Science Support Center. The standard extractive work-up procedure consisted of pouring into a large amount of water, extracting thoroughly with the organic solvent indicated, washing the combined extract successively with water and brine, drying the extract over anhydrous Na_2SO_4 or $MgSO_4$, and evaporating the solvent.

1-*O*-benzoyl-(2,3:5,6)-di-*O*-isopropylidene-*scyllo*-inositol (5a) and 1-*O*-benzoyl-(2,3:4,5)-di-*O*-isopropylidene-*scyllo*-inositol (5b).

To a solution of compound **4** [1] (6g, 21.11 mmol) and p-TSA (400mg, 2.1 mmol) in dry DMF (100 mL) at RT, was slowly added 2-methoxypropene (20 mL, 211.2 mmol) over 30 min. After stirring for 24h the reaction mixture was poured into aq. $NaHCO_3$ with vigorous agitation and extracted with EtOAc. The organic layer was dried over anhydrous $MgSO_4$, filtered and concentrated. The crude two regio-isomeric products were isolated by recrystallization, and column chromatography on silica gel gave compound **5a** (4.3g, 51%) and **5b** (2.1g, 30%), both as white solid. **5a**: R_f 0.26 (EtOAc : n-Hex = 1:2); mp. 284-286 ^{0}C; ^{1}H NMR ($CDCl_3$): δ 1.44, 1.48 (s, 6H), 2.46 (d, *J* = 2.8 Hz, 1H), 3.76 (app. t, *J* = 9.4 Hz, 2H), 3.88 (app. t, *J* = 9.4 Hz, 2H), 4.13 (dt, *J* = 2.8, 8.8 Hz, 1H), 5.7 (t, *J* = 9.3 Hz, 1H), 7.27-8.1 (m, 5H). **5b**: R_f 0.4 (EtOAc : n-Hex = 1:2); mp. 206-207 ^{0}C; ^{1}H NMR ($CDCl_3$): δ 1.47-1.48 (s, 6H), 1.49-1.51 (s, 6H), 2.88 (d, *J* = 4.1 Hz, 1H), 3.67-3.9 (m, 4H), 4.13 (m, 1H), 5.43 (dd, *J* = 7.9, 10.5 Hz, 1H), 7.45-8.11 (m, 5H);

4-*O*-Benzoyl-1-*O*-*t*-butyldimethylsilyl-(2,3:5,6)-di-*O*-isopropylidene-*scyllo*-inositol (6a) and 2-*O*-Benzoyl-1-*O*-*t*-butyldimethylsilyl-(3,4:5,6)-di-*O*-isopropylidene-*scyllo*-inositol (6b). TBDMSCl (534 mg, 3.54 mmol) was added to a solution of compounds **5a** and **5b** (646 mg, 1.77 mmol) and imidazole (534 mg, 3.54 mmol) in DMF (3 mL) at 0 ^{0}C, and the mixture was stirred for 10h at RT. The mixture was diluted with EtOAc, washed with water and concentrated under vacuum. The crude product was purified by flash chromatography (silica gel, hex: EtOAc 3: 1) to afford a mixture of **6a** and **6b** as white solid. Yield = 750 mg, 88%. ^{1}H NMR ($CDCl_3$): δ -0.07, 0.06 (2s, 6H, for **6a**), 0.14 (s, 6H, for **6b**), 0.73 (s, 9H, for **6a**), 0.92 (s, 9H, for **6b**), 1.41-1.46 (m, 12H), 3.60-4.10 (m, 5H), 5.47-5.56 (m, 1H), 7.41-7.46 (m, 2H), 7.54-7.56 (m, 1H), 8.00-8.09 (m, 2H). ^{13}C NMR ($CDCl_3$): δ -4.8, -4.5, -4.3, 18.1, 25.6, 25.9, 26.7, 26.83, 26.86, 26.9, 26.95, 70.0, 73.1, 74.7, 75.6, 76.1, 78.4, 79.3, 81.2, 81.9, 112.8, 113.3, 128.4, 130.1, 130.15, 130.2, 133.2, 165.5; HRMS (FAB): m/z calcd. for $C_{25}H_{38}O_7Si$: 478.2465; found 479.2460 $[M+H]^+$.

1-*O*-*t*-butyldimethylsilyl-(2,3:5,6)-di-*O*-isopropylidene-*scyllo*-inositol (7a) and 1-*O*-*t*-butyldimethylsilyl-(2,3:4,5)-di-*O*-isopropylidene-*scyllo*-inositol (7b).
NaOMe (134 µL, 0.58 mmol, 25 wt %) was added to a solution of compounds **6a** and **6b** (700 mg, 1.46 mmol) in dichloromethane: MeOH (1:4, 2.5 mL), and the solution was refluxed for 3h. After cooling to RT, the reaction mixture was diluted with dichloromethane and filtered through silica gel. Filtrate was concentrated and the residue washed with 5% EtOAc in hexane to remove the byproduct methyl benzoate. The product was dried under vacuum to afforded **7a** and **7b** as white solid. Yield = 547 mg, quantitative. ^{1}H NMR ($CDCl_3$): δ 0.07-0.13 (m, 6H), 0.85-0.90 (m, 9H), 1.40-1.46 (m, 12H), 3.43-3.78 (m, 6H); HRMS (FAB): m/z calcd. for $C_{18}H_{34}O_6Si$: 374.2203; found 375.2200 $[M+H]^+$.

1-*O*-*t*-butyldimethylsilyl-4-*O*-(6-benzoyloxycarbonyl-aminohexanoyl)-(2,3:5,6)-di-*O*-isopropylidene-*scyllo*-inositol (8a) and 1-*O*-*t*-butyldimethylsilyl-2-*O*-(6-benzoyloxycarbonyl-aminohexanoyl)-(3,4:5,6)-di-*O*-isopropylidene-*scyllo*-inositol (8b). Compound **7a** and **7b** (360 mg, 0.960 mmol), 6-benzoyloxycarbonyl-aminohexanoic acid (290 mg, 1.15 mmol), EDC (221 mg, 1.15 mmol) and DMAP (35 mg, 0.288 mmol) were dissolved in CH_2Cl_2 (3 mL) and solution was stirred for 24h at RT under N_2. The reaction mixture was diluted with EtOAc, washed with H_2O, $NaHCO_3$ and brine. The organic phase was separated, dried over Na_2SO_4 and concentrated. The crude product was purified by column chromatography (EtOAc: n-Hex = 1:3) to afford compounds **8a** and **8b** (485 mg, 81 %) as sticky liquid. ^{1}H NMR

($CDCl_3$): δ 0.05 (s, 3H, for **8a**), 0.08 (s, 3H, for **8a**), 0.11 (s, 6H, for **8b**), 0.84 (s, 9H, for **8a**), 0.90 (s, 9H, for **8b**), 1.37-1.53 (m, 16H), 1.63-1.68 (m, 2H), 2.32-2.39 (m, 2H), 3.15-3.19 (m, 2H), 3.50-3.62 (m, 2H), 3.63-3.68 (m, 1H), 3.71-3.79 (m, 1H), 3.89 (dd, *J* = 7.8, 10.0 Hz, 1H), 4.81 (br s, 1H), 5.08 (s, 2H), 5.22 (dd, *J* = 7.8, 10.7 Hz, 1H), 7.30-7.36 (m, 5H); ^{13}C NMR ($CDCl_3$): δ -4.7, -4.4, -4.1, 18.3, 18.7, 24.5, 24.6, 25.9, 26.0, 26.2, 26.4, 26.8, 26.9, 27.0, 27.05, 29.9, 34.4, 41.2, 66.9, 69.5, 70.1, 72.9, 74.1, 75.7, 76.1, 78.4, 79.5, 81.3, 112.9, 113.4, 113.9, 128.4, 128.8, 136.9, 156.7, 172.5, 172.6; HRMS (FAB): m/z calcd. for $C_{32}H_{51}O_9NSi$: 621.3411; found 622.3410 $[M+H]^+$.

1-*O*-(6-benzoyloxycarbonyl-aminohexanoyl)-*scyllo*-inositol (9).

To a solution of compound **8a** and **8b** (310 mg, 0.5 mmol) in CH_2Cl_2: MeOH (1: 4, 2mL), was added HCl (g)-saturated EtOAc (1 mL), and stirred at RT for 30 min. After evaporation of solvents the residue was washed with hexane and ether, dried under vacuum to afford compound **9** (225 mg, 91%) as white solid. mp = 204-205 ^{0}C; ^1H NMR (D_2O): δ 1.33-1.37 (m, 2H), 1.48-1.53 (m, 2H), 1.62-1.67 (m, 2H), 2.46 (t, *J* = 7.1 Hz, 2H), 3.12 (t, *J* = 6.0 Hz, 2H), 3.34-3.50 (m, 6H), 5.12 (s, 2H), 7.42-7.44 (m, 5H); HRMS (FAB): m/z calcd. for $C_{20}H_{29}NO_9$: 427.1921; found 428.1920 $[M+H]^+$.

1-*O*-(6-benzyloxycarbonyl-aminohexanoyl)-2,3,4,5,6-penta-*O*-(*N*,*N*'-di-Boc-N"-aminohexanoylguanidine)-*scyllo*-inositol (10).

A mixture of compound **9** (85.5 mg, 0.2 mmol), Acid-A (598 mg, 1.6 mmol), EDC (306 mg, 1.6 mmol), and DMAP (49mg, 0.4 mmol) in CH_2Cl_2 (4 mL) was stirred at RT under N_2 for 24h. The reaction mixture was diluted with CH_2Cl_2, washed several times with H_2O, $NaHCO_3$ and brine. The organic phase was separated, dried over Na_2SO_4 and concentrated. The crude product was purified by column chromatography on silica gel (EtOAc: Hex = 1:1) to give compound **10** (402 mg, 91%) as stick solid. R_f 0.38 (EtOAc: Hex = 1:1); ^1H NMR ($CDCl_3$): δ 1.23-1.68 (m, 126H), 2.19-2.22 (m, 12H), 3.1-3.18 (m, 2H), 3.36-3.40 (m, 10H), 5.10 (s, 2H), 5.24-5.32 (m, 6H), 5.25 (s, 5H), 7.33 (br s, 5H), 8.29 (br s, 5H), 11.50 (s, 5H); ^{13}C NMR ($CDCl_3$) 24.4 , 24.5, 26.2, 26.4, 27.2, 27.8, 28.1, 28.3, 28.6, 28.7, 29.7, 33.7, 40.6, 40.7, 66.4, 69.9, 77.4, 79.1, 79.2, 83.0, 83.1, 128.0, 128.4, 136.8, 153.3, 156.1, 156.5, 163.5, 163.6, 171.8; MALDI-TOF MS: m/z calcd. for $C_{105}H_{174}N_{16}O_{34}$: 2203.2; found: 2204.8 $[M+ H]^+$.

1-*O*-(6-aminohexanoyl)-2,3,4,5,6-penta-*O*-(*N*,*N*'-di-Boc-N"-aminohexanoyl-guanidine)-*scyllo*-inositol (11).

Solution of compound **10** (100 mg, 0.045 mmol) in a mixed solvent of CH_2Cl_2: MeOH (1:9, 5 mL) was hydrogenated (40 psi) at RT over 10% Pd/C (25 mg). After 12h, the catalyst was filtered through celite bed and the filtrate was evaporated to afford free amine **11** (87 mg, 92%) as sticky solid. ^{1}H NMR ($CDCl_3$): δ 1.25-1.56 (m, 126H), 2.21 (t, *J* = 6.8, 12H), 2.92-2.99 (m, 2H), 3.36-3.48 (m, 10H), 5.23 (s, 6H), 8.29-8.36 (m, 5H), 11.5 (m, 5H); MALDI-TOF MS: m/z calcd. for $C_{97}H_{168}N_{16}O_{32}$: 2069.2; found: 2070.3[M+ H]$^+$.

1-*O*-[6-(*N*-fluoresceinyl-5-thioureido)-hexanoyl]-2,3,4,5,6-penta-*O*-(*N*,*N*'-di-Boc-N"-aminohexanoyl -guanidine)-*scyllo*-inositol (12).

To a solution of **11** (85 mg, 0.04 mmol) in a mixed solvent THF and abs. ethanol (5 mL, 3:2), were added fluorescein-5-isothiocyanate (19.1 mg, 0.049 mmol) and triethylamine (17.1 µL, 0.123 mmol). The reaction mixture was stirred for 24h at RT in dark. Reaction mixture was concentrated, diluted with ethyl acetate, and washed with water. Organic phase was separated, dried over Na_2SO_4 and concentrated. The crude product **12** (103 mg) was directly used for the next step without further purification. ^{1}H NMR ($CDCl_3$): δ 1.23-1.49 (m, 126H), 2.11-2.25 (m, 12H), 3.05-3.75 (m, 12H), 5.24 (s, 6H), 6.55-7.11 (m, 6H), 7.95-8.17 (m, 3H), 8.35-8.41 (m, 5H), 11.5 (m, 5H).

1-*O*-[6-(*N*-fluoresceinyl-5-thioureido)-hexanoyl]-2,3,4,5,6-penta-*O*-(aminohexanoyl guanidinium)-*scyllo*-inositol (2).

A solution of **12** (103 mg, crude) in HCl (g)-saturated ethyl acetate (2 mL) was stirred at RT for 24h. The solution was concentrated, repeatedly washed with ethyl acetate and ether, and the crude product was purified by MPLC with Fluka 100 C8-reversed phase silica gel (CH_3CN:H_2O = 1:1, containing 0.1% TFA in both CH_3CN and H_2O). Freeze drying afforded the protonated form of **2** as greenish yellow foamy solid (42 mg, 61% in two steps). UV (H_2O): $\lambda_{max}(\varepsilon)$ = 481 nm (27502 cm^{-1} M^{-1}); ^{1}H NMR (CD_3OD): δ 1.23-1.38 (m, 12H), 1.58-1.72 (m, 24H), 2.23-2.32 (m, 12H), 3.06-3.15 (m, 10H), 3.61 (m, 1H), 3.94 (m, 1H), 5.43 (s, 6H), 6.54-6.74 (m, 6H), 7.15-7.18 (m, 1H), 7.70-7.73 (m, 1H), 8.01-8.25 (m, 1H); MALDI-TOF MS: m/z calcd. for $C_{68}H_{99}N_{17}O_{17}S$: 1457.7; found: 1457.8 [M]$^+$. Analytical HPLC: t_R = 2.819 min (ZORBAX SB-C8, flow rate 1 mL/min; UV: λ = 220 nm; H_2O: CH_3CN = 50: 50 with 0.1% TFA), purity > 95%.

Scheme 2 Synthesis of Acid-A

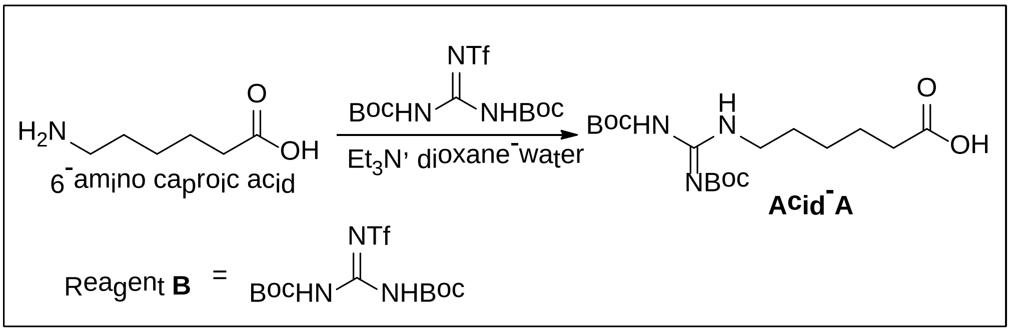

6-(2,3-bis(tert-butoxycarbonyl)guanidino)hexanoic acid (Acid-A).

6-Amino caproic acid (3g, 22.87 mmol) was dissolved in 60 mL of water and 140 mL of 1,4-dioxane. Et_3N (7.9mL, 57.17 mmol) was added, and the solution was stirred for 5min at RT. To this solution, was added N,N'-di-Boc-N''-triflylguanidine (Reagent **B**, 13.4g, 34.3 mmol).[2] The reaction mixture was stirred for 3 days at room temperature before removing dioxane under reduced pressure. The residue was diluted with EtOAc, and the organic phase washed with saturated NH_4Cl solution. The crude product was purified by column chromatography (230-400 mesh size silica gel) using MeOH and DCM system, followed by trituration with hexane to obtain the desired Acid-A (5.2g, 60%) as white solid. R_f 0.3 (10% MeOH in DCM); ^{1}H NMR ($CDCl_3$): 11.46 (br s, 1H), 8.34 (br s, 1H), 3.42 (q, J = 6.5 Hz, 2H), 2.36 (t, J = 7.5 Hz, 2H), 1.7 – 1.39 (m, 24H); ^{13}C NMR ($CDCl_3$): 180.8, 165.28, 158.0, 155.1, 84.9, 81.1, 42.5, 35.8, 30.5, 30.1, 29.9, 28.1, 26.2.

Scheme 3 Synthesis of BBB-permeable *scyllo*-Inositol derivative (AAD-66) **3**

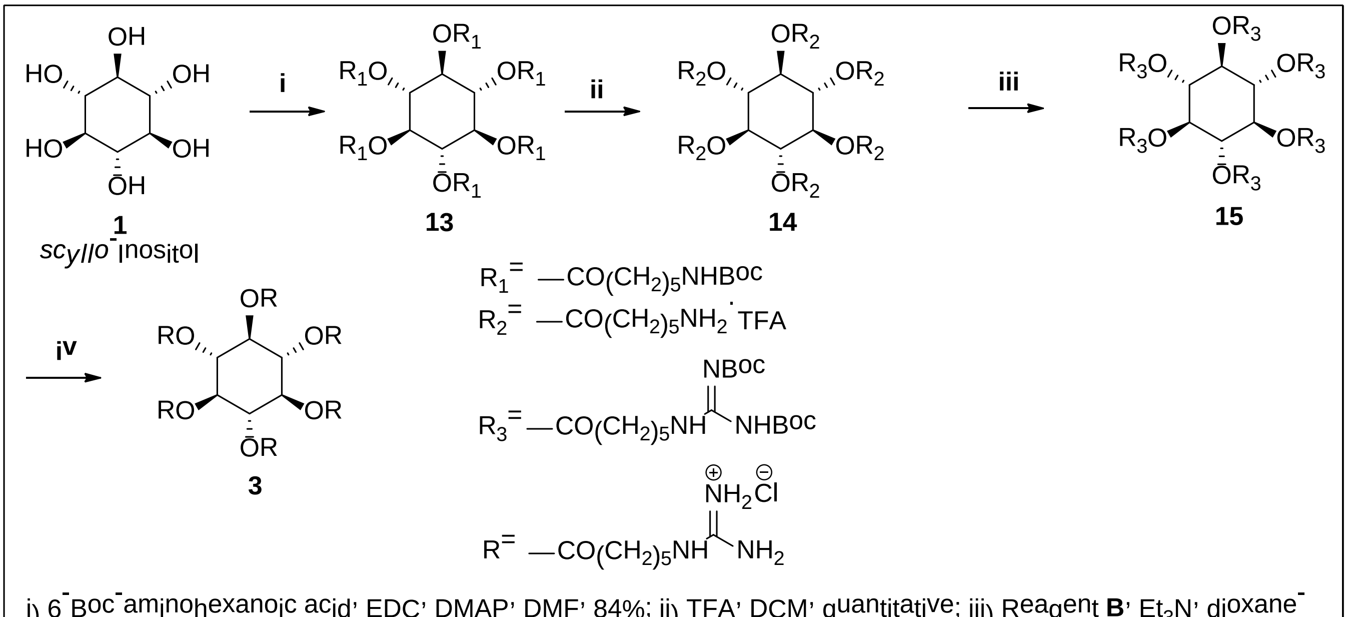

i) 6-Boc-aminohexanoic acid, EDC, DMAP, DMF, 84%; ii) TFA, DCM, quantitative; iii) Reagent **B**, Et_3N, dioxane-water-DCM, 95%; iv) HCl(g), EtOAc, rt, 80%.

Hexa-*O*-(6-Boc-aminohexanoyl)-*scyllo*-inositol (13).

A mixture of *scyllo*-inositol (**1**, 118 mg, 0.195 mmol), 6-Boc-aminohexanoic acid (270 mg, 1.17 mmol), EDC (224 mg, 1.17 mmol) and DMAP (28.5 mg, 0. 234 mmol), dry DMF (1.5 mL) was stirred at RT under N_2-atmosphere for 24h. The reaction mixture was diluted with EtOAc, washed with saturated $NaHCO_3$ several times to remove the unreacted acid. The organic phase was separated, dried over Na_2SO_4 and concentrated. The crude residue was purified by column chromatography (EtOAc: n-Hex = 1:2 – 2:3) to give compound **13** (240 mg, 84.5%) as white foamy solid. R_f 0.32 (EtOAc: n-Hex = 1:1); ^{1}H NMR ($CDCl_3$): 1.19-1.57 (m, 90H), 2.20 (t, J = 7.5 Hz, 12H), 3.05-3.11 (m, 12H), 4.85 (br s, 6H), 5.27 (s, 6H); ^{13}C NMR ($CDCl_3$): δ 24.6, 26.4, 28.6, 29.9, 34.0, 40.5, 70.1, 79.2, 156.2, 172.1.

Hexa-*O*-(6-aminohexanoylhydrochloride)-*scyllo*-inositol (14).

To a solution of compound **13** (200 mg, 0.137 mmol) in CH_2Cl_2 (2.5 mL), was added dropwise 0.5 mL TFA and the solution was stirred at RT for 6h. The reaction mixture was concentrated, dried under vacuum, and washed with ether to yield compound **14** (198 mg, quantitative) as white sticky solid. ^{1}H NMR (CD_3OD): δ 1.37 -1.42 (m, 12H), 1.52-1.69 (m, 24H), 2.27 (t, J = 7.5 Hz, 12H), 2.91 (t, J = 7.6 Hz, 12H), 5.43 (s, 6H); ^{13}C NMR (CD_3OD): δ 25.5, 27.1, 28.4, 34.8, 40.6, 71.8, 173.5.

Hexa-*O*-(*N*,*N'*-bis-Boc-*N''*-aminohexanoylguanidine)-*scyllo*-inositol (15).

To a solution of compound **14** (100 mg, 0.069 mmol) in a mixed solvent, 1, 4 dioxane: H_2O: CH_2Cl_2 (5: 1: 2), was added TEA (0.115 ml, 0.828 mmol) and Reagent **B** (324 mg, 0.828 mmol), and the solution was stirred for 48h at RT. The reaction mixture was concentrated and extracted with EtOAc. The organic phase was washed with water and brine, dried over Na_2SO_4 and concentrated. The crude product was purified by column chromatography (EtOAc: n-Hex = 1:1 – 2:1) to afford compound **15** (150 mg, 95%) as white foamy solid. R_f 0.61 (EtOAc: n-Hex = 2:3); ^{1}H NMR ($CDCl_3$): δ 1.24-1.70 (m, 144H), 2.17-2.22 (m, 12H), 3.33-3.40 (m, 12H), 5.22 (s, 6H), 8.29 (s, 6H), 11.45 (s, 6H); ^{13}C NMR ($CDCl_3$): δ 24.8, 26.7, 28.4, 28.6, 29.0, 34.0, 40.9, 70.2, 79.5, 83.4, 153.6, 156.4, 163.8, 172.1.

The present reaction was also carried out in a different solvent system of MeOH: CH_2Cl_2 (4: 1) to afford a similar yield.

Hexa-*O*-(aminohexanoylguanidine)-*scyllo*-inositol·6HCl (3).

A solution of compound **15** (150 mg, 0.065 mmol) in HCl(g)-saturated EtOAc (2 mL) was stirred at RT for 24 h. Solvent was evaporated to dryness under vacuum and the residue was washed with EtOAc and ether several times. The residue was dissolved in deionized water, filtered through a PTGE syringe filter and lyophilized to provide compound **3** (69 mg, 80%) as white foamy solid. For analytical sample the residue was purified by using semi preparative HPLC (Agilant 1100 chemstation) on VYDAC-C18 monochromatic reverse phase column (100% to 10% acetonitrile in water for 55min, 2.7 mL/ min flow rate: UV: λ = 220 nm). ^{1}H NMR (CD_3OD): δ 1.34-1.40 (m, 12H), 1.55-1.61 (m, 24H), 2.27 (t, *J* = 7.3 Hz, 12H), 3.18 (t, *J* = 7.0 Hz, 12H), 5.43 (s, 6H); ^{13}C NMR (CD_3OD): δ 25.7, 27.4, 29.7, 34.9, 42.4, 71.7, 158.8, 173.6; MS (MALDI-TOF): m/z calcd. for $C_{48}H_{90}N_{18}O_{12}$:1110.71; found: 1110.699 (M^+).

Analytical HPLC: t_R = 2.45 min (KROMASIL-C8, flow rate 1 mL/ min; UV: λ = 220 nm; H_20: CH_3CN 70: 30 with 0.1% HCl), purity > 98%.

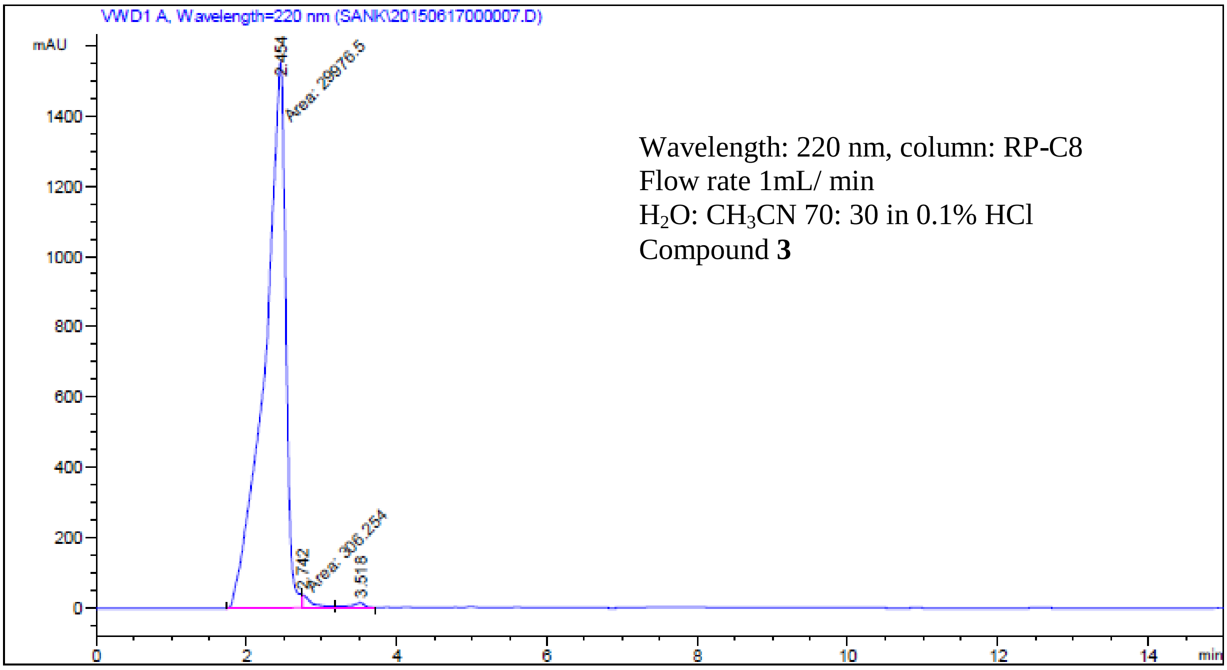

MALDI TOF data for compound **3**

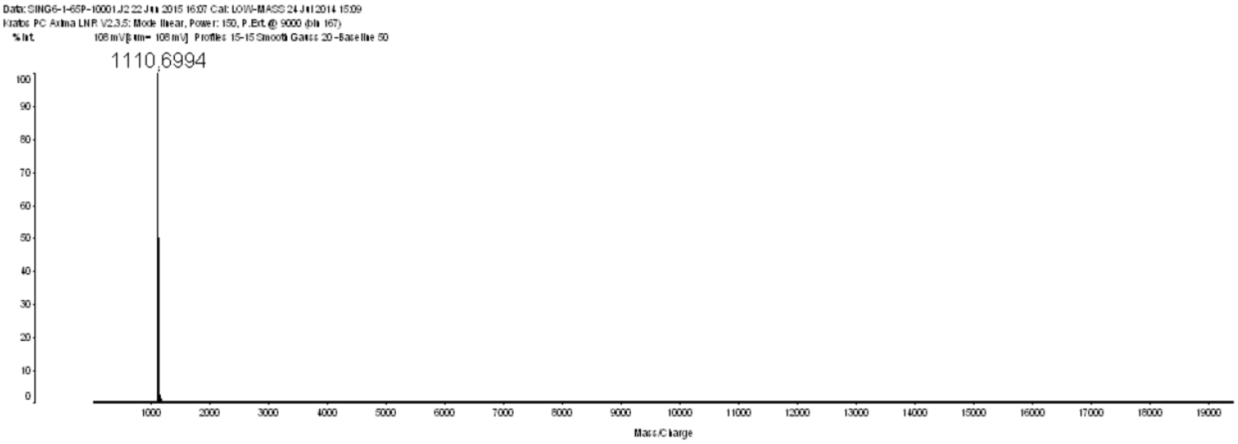

^{1}H NMR of compound no **3**

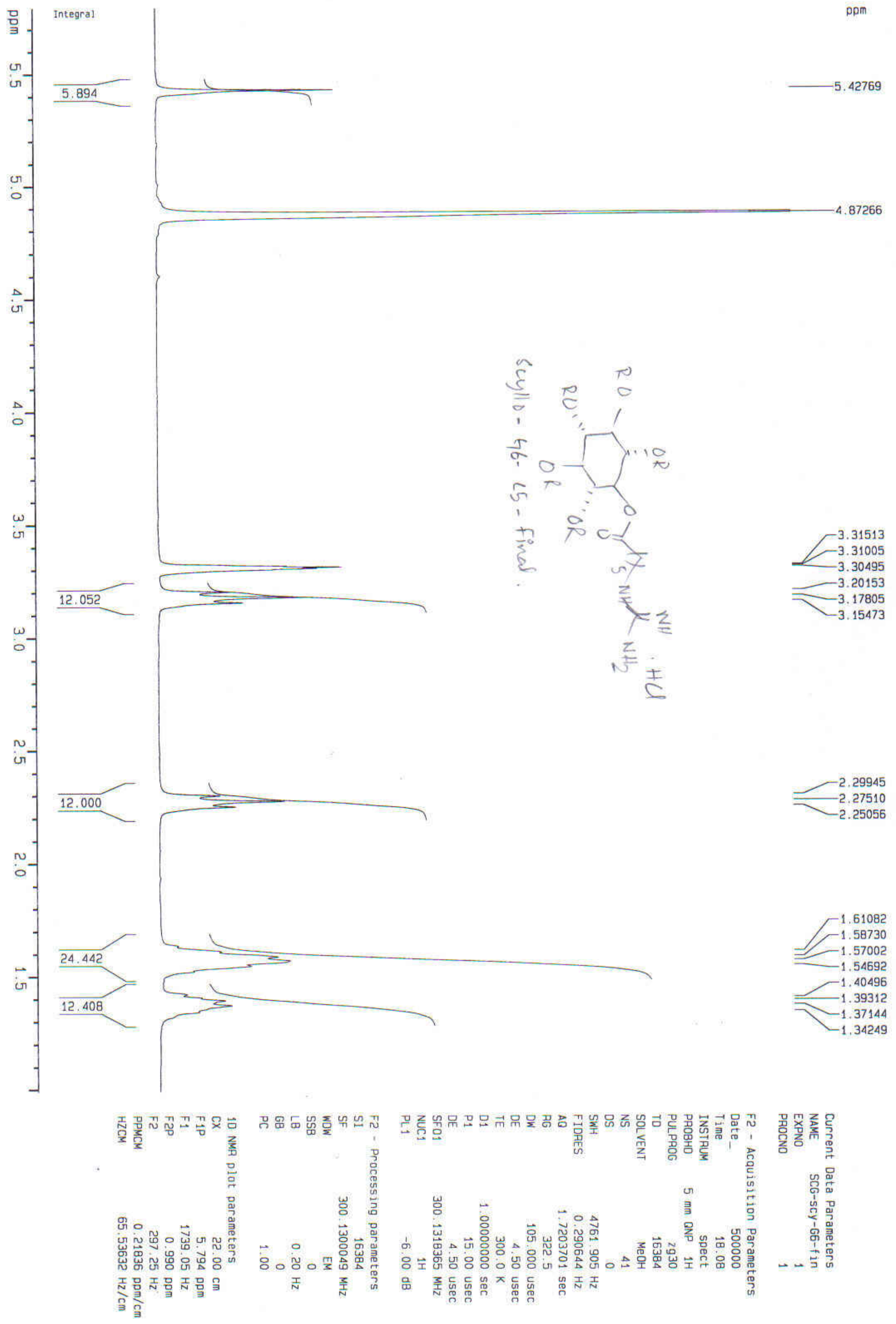

^{13}C NMR of compound no **3**

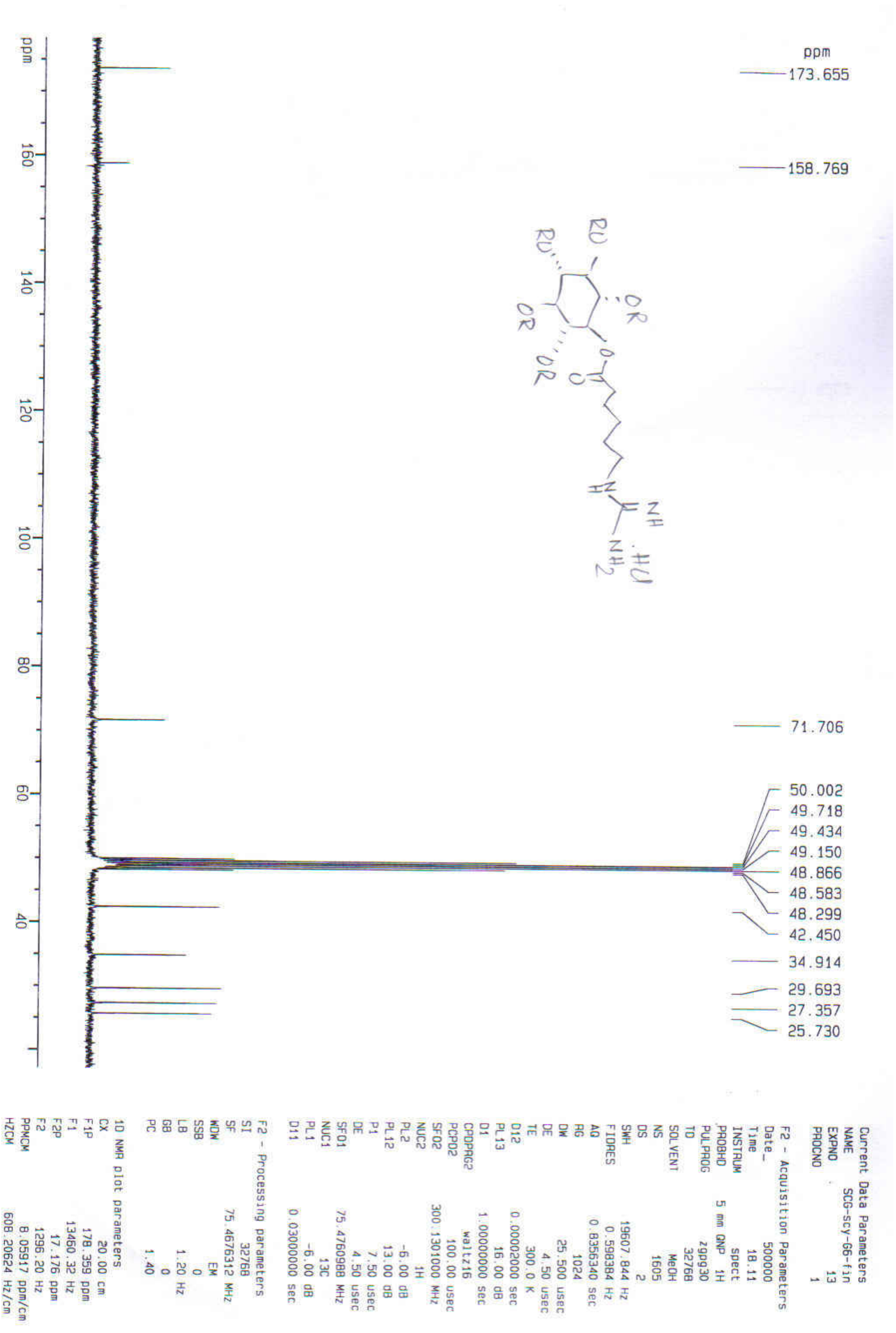

Supplementary References

1. Chung S.K., *et al.* Synthesis of all possible regioisomers of scyllo-inositol phosphate. *Bioorg Med Chem* **7**, 2577-2589 (1999).
2. Wu C.H., *et al.* Attenuating HIV Tat/TAR-mediated protein expression by exploring the side chain length of positively charged residues. *Org Biomol Chem* **13**, 11096-11104 (2015).
